# Effects of Tumor-Rib Distance and Dose-Dependent Rib Volume on Radiation-Induced Rib Fractures in Patients with Breast Cancer

**DOI:** 10.3390/jpm12020240

**Published:** 2022-02-08

**Authors:** Sang Mi Lee, Jeong Won Lee, Woo Chul Kim, Chul Kee Min, Eun Seog Kim, In Young Jo

**Affiliations:** 1 Department of Nuclear Medicine, Soonchunhyang University Cheonan Hospital, 31 Suncheonhyang 6-gil, Dongnam-gu, Cheonan 31151, Korea; c91300@schmc.ac.kr; 2 Department of Nuclear Medicine, International St. Mary’s Hospital, College of Medicine, Catholic Kwandong University, Simgok-ro 100-gil 25, Seo-gu, Incheon 22711, Korea; sads00@naver.com; 3 Department of Radiation Oncology, Soonchunhyang University Cheonan Hospital, 31 Suncheonhyang 6-gil, Dongnam-gu, Cheonan 31151, Korea; veura.wc@gmail.com (W.C.K.); arnoldmin74@gmail.com (C.K.M.); radio@schmc.ac.kr (E.S.K.)

**Keywords:** breast cancer, radiation therapy, rib fracture, bone scintigraphy

## Abstract

This study aimed to investigate the effects of tumor-rib distance and dose-dependent rib volume on radiation-induced rib fractures (RIRFs) in patients with breast cancer. We retrospectively included 510 women with breast cancer who underwent surgical resection with adjuvant radiotherapy. The tumor-rib distance was measured using preoperative computed tomography (CT) images. Postoperative chest wall thickness and dose-dependent rib volumes, which are absolute rib volumes receiving >20 Gy (V20), 30 Gy (V30), 40 Gy (V40), 45 Gy (V45), and 50 Gy (V50), were measured from the stimulation CT images for radiation treatment planning. We assessed the relationship of RIRF with tumor-rib distance, postoperative chest wall thickness, and dose-dependent rib volumes. Patients with high values of tumor-rib distance and postoperative chest wall thickness had significantly lower risks of RIRF than those with low values. Patients with high values of V20, V30, V40, V45, and V50 had significantly higher risks of RIRF than those with low values. In a multivariate analysis, tumor-rib distance and all five dose-dependent rib volumes, as well as osteoporosis and radiation field, were independent risk factors for RIRF. Tumor-rib distance and dose-dependent rib volume were independent risk factors for RIRF in patients with breast cancer.

## 1. Introduction

Worldwide, breast cancer has the highest incidence and mortality rates among cancers in women [1]. Resection of primary breast cancer lesions with surgical axillary staging is the standard curative treatment for patients with breast cancer [2]. For patients treated with either breast conservative surgery or mastectomy, adjuvant radiotherapy is recommended to eradicate any remaining tumor cells [2]. Adjuvant radiotherapy has an established crucial role in the curative management of patients with breast cancer, showing survival benefits of reducing recurrence rates and improving overall survival in a meta-analysis study including more than 10,000 patients [3,4]. However, adjuvant radiotherapy also involves several complications that have been receiving increased attention considering increased survival periods among patients with breast cancer [5,6]. Radiation-induced rib fracture (RIRF) is one of the well-known adverse effects of conventional radiotherapy in patients with breast cancer, with an occurrence rate of 1.0–18.5% [4,7,8,9,10]. Although most patients with RIRF show no or minimal symptoms, several patients experience long-lasting moderate pain and receive analgesic treatment for pain relief [5,9,11]. Furthermore, RIRF is characterized by abnormal findings on bone scintigraphy, which is often used for follow-up surveillance of patients with breast cancer, and these abnormal findings can be misdiagnosed as bone metastasis [9,10,12]. Therefore, several studies have tried to identify the risk factors for RIRF among radiotherapy-related factors and clinical factors [7,8,9,12].

Radiation-induced biological changes in bones are known to be dose-dependent [13,14]. In previous studies with lung cancer patients, the risk of chest wall complications, including RIRF, was significantly associated with the dosimetric parameters of radiation to the ribs [13,15]. The distance from the tumor to the rib (tumor-rib distance) and the absolute rib volume receiving a certain radiation dose were significant risk factors for RIRF in patients with lung cancer [13]. Given the physical proximity between breast tissue and ribs, the risk of RIRF in patients with breast cancer could show a significant relationship with the dosimetric parameters of radiation to the ribs, which is similar to the aforementioned findings in the studies with lung cancer [13,15]. However, the effect of tumor-rib distance and dose-dependent rib volume on the risk of RIRF in patients with breast cancer remains unclear.

Thus, this study aimed to investigate the relationship of tumor-rib distance, postoperative chest wall thickness, and dose-dependent rib volume on computed tomography (CT) images with the risk of RIRF in patients with breast cancer who received adjuvant radiotherapy after curative resection.

## 2. Materials and Methods

### 2.1. Study Population

We retrospectively reviewed medical records of female patients with histopathologically diagnosed breast cancer who underwent curative breast surgery and subsequent adjuvant radiotherapy at our medical center from January 2011 to December 2017. Among them, we finally enrolled 510 patients who underwent bone scintigraphy for staging and surveillance and in whom no bone metastasis was found during follow-up. The exclusion criteria were as follows: the patients (1) who had distant metastasis on staging work-up examinations, (2) who showed bone metastasis on follow-up imaging studies, (3) who had a history of radiotherapy in the breast or chest wall owing to metachronous breast cancer, (4) who had a history of other malignant diseases, and (5) who were lost to follow-up within 24 months after radiotherapy.

All patients underwent staging work-up examinations, including breast ultrasonography, contrast-enhanced chest CT, and bone scintigraphy. Based on staging examination findings, breast-conserving surgery or total mastectomy with/without neoadjuvant chemotherapy was performed. After surgery, adjuvant radiotherapy with/without chemotherapy and/or hormone therapy was performed based on histopathological results and the clinical condition of patients.

### 2.2. Radiotherapy

All patients underwent simulation CT using the Philips Brilliance Big Bore (Philips Medical Systems, Cleveland, OH, USA) with a 3-mm slice thickness. Patients were placed in the supine position on a no-tilting breast board (CIVCO Medical Solutions, Orange City, IA, USA) with their arms above the head for appropriate exposure of the breasts and axillae. Patients treated for ipsilateral supraclavicular lymph nodes turned their heads to the contralateral side to reduce radiation-induced side effects. All patients underwent simulation CT with free-breathing. The treatment targets were delineated based on the Radiation Therapy Oncology Group contouring guidelines [16]. On the basis of the guidelines, the treatment targets included the whole breast after breast-conserving surgery or the chest wall, including the ribs, after total mastectomy. If necessary, regional lymph nodes (i.e., supraclavicular and/or internal mammary lymph nodes) were included as treatment targets based on pathologic staging. Treatment planning was performed using a radiotherapy treatment planning system (Eclipse ver. 8.9 (Varian Medical Systems, Palo Alto, CA, USA) or Monaco ver. 5.11 (Elekta Oncology System, Crawley, UK)) with a 6-MV photon. For all patients, treatment was delivered in two steps to the whole breast and tumor bed boost. The dose schemes involved administration to the breast at 50 Gy in 25 fractions for 5 weeks, followed by administration to the tumor bed boost of 10-16 Gy in 5–8 fractions based on the resection margin status. All enrolled patients were treated with three-dimensional conformal radiotherapy (3D-CRT) or volumetric modulated arc therapy (VMAT) using linear accelerator system machines.

### 2.3. Follow-Up and RIRF Assessment

Before adjuvant treatment, all enrolled patients underwent dual-energy X-ray absorptiometry to determine bone mineral density. Osteoporosis was defined as bone mineral density ≤2.5 standard deviations below the mean bone mineral density of young healthy women. Osteopenia was defined as bone mineral density of 1.0–2.5 standard deviations below the aforementioned mean.

After curative surgery, follow-up bone scintigraphy was performed every 6 months in the first 2 years, followed by every 12 months. All enrolled patients were followed up with at least four bone scans. Two nuclear medicine physicians retrospectively reviewed bone scan images, with between-reader discrepancies being resolved through consensus reading. In patients who newly showed abnormally increased radiotracer uptake in the ribs on follow-up bone scans, rib lesions were categorized as a benign rib fracture and bone metastasis based on additional imaging studies, including CT, F-18 fluorodeoxyglucose positron emission tomography (PET)/CT, and follow-up bone scans. Benign rib fracture was defined as rib lesions showing a simple fracture on CT and/or PET/CT or revealing gradually decreased uptake on serial follow-up bone scans without any treatment. Bone metastasis was defined as a rib lesion showing bone destruction on CT and/or PET/CT images or increased intensity and extent of radiotracer uptake, with an increased number of bone lesions suggestive of bone metastasis on follow-up bone scans. In patients with benign rib fractures on bone scintigraphy images, radiotherapy simulation CT images were further reviewed to determine whether rib lesions were included in the irradiated field. The benign rib fracture lesions identified in the irradiated field were defined as RIRF. Even though benign rib fracture lesions were located within the irradiated field, the rib lesions were excluded from RIRF if the patients had a history of traumatic events in the chest wall area, including traffic accidents, serious falls, or sports injuries. RIRF detected using bone scintigraphy was considered as an event in the analysis. Patients in whom bone metastasis was detected during follow-up examinations were excluded from the analysis, and those diagnosed with benign rib fractures other than RIRF were included in the study but were determined to have no event.

### 2.4. Imaging Analysis

**Figure 1:**
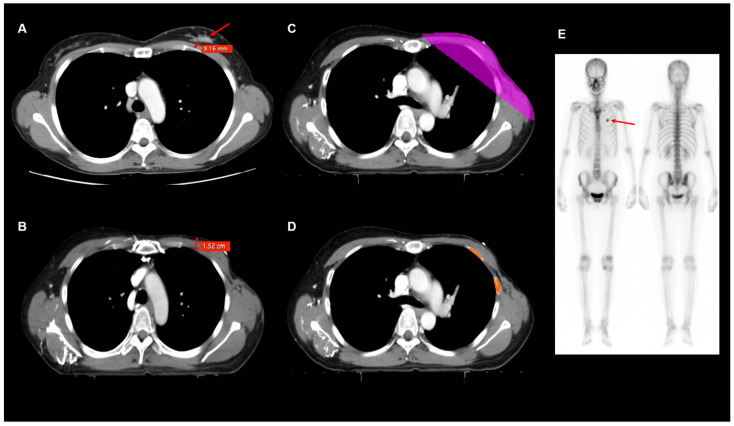
Preoperative contrast-enhanced CT image (**A**), radiotherapy simulation contrast-enhanced CT images (**B**–**D**), and bone scintigraphy images (**E**) showing an example of measurement of the tumor-rib distance, postoperative chest wall thickness, and V40 in a 43-year-old woman with left breast cancer. On preoperative CT images, the minimum distance from the margin of the primary tumor (arrow) to the rib was measured (0.9 cm), which was defined as the tumor-rib distance (**A**). On postoperative radiotherapy simulation CT images, the soft tissue thickness of the chest wall in the mid-clavicular line at the levels of the 2nd intercostal space (1.5 cm) was measured, as well as the 3rd and 4th intercostal spaces (**B**). Within the isodose area of 40 Gy (**C**), an area with a CT-attenuation range of 200–2000 HU in the ribs was selected (**D**), and the total volume of selected ribs was defined as the absolute rib volume receiving >40 Gy (V40), which was 41.0 cm^3^. On follow-up bone scintigraphy images (**E**) obtained 24.2 months after radiotherapy, RIRF was found in the anterior arc of the left 3rd rib (arrow).

Seven imaging parameters were measured for each patient: tumor-rib distance; postoperative chest wall thickness; and five dose-dependent rib volumes, which represented absolute rib volumes receiving >20 Gy (V20), 30 Gy (V30), 40 Gy (V40), 45 Gy (V45), and 50 Gy (V50) (Figure 1). The tumor-rib distance was measured using contrast-enhanced CT images obtained for the staging work-up. The tumor-rib distance was defined as the minimum distance between the primary tumor margin and the rib. Postoperative chest wall thickness was measured from radiotherapy simulation CT images. The soft tissue thickness of the chest wall in the mid-clavicular line at the levels of the 2nd, 3rd, and 4th intercostal space was measured; moreover, the mean value of chest wall thickness at those three levels was defined as the postoperative chest wall thickness [17,18]. Five volumetric parameters of the absolute rib volume were measured using radiotherapy simulation CT images with the open-source LIFEx software version 7.0.0 (www.lifexsoft.org) [19]. The total volume of areas with a CT-attenuation range between 200 Hounsfield unit (HU) and 2000 HU in the ribs within the 20 Gy, 30 Gy, 40 Gy, 45 Gy, and 50 Gy isodose areas on radiotherapy simulation CT images were defined as V20, V30, V40, V45, and V50, respectively. Bony structures other than the ribs were manually removed; accordingly, only the total volumes of the ribs within the isodose areas were measured to obtain those five volumetric parameters.

### 2.5. Statistical Analysis

The Mann–Whitney test was performed to compare the seven imaging parameters between patients with RIRF and without RIRF, between patients who received irradiation only in the whole breast and in the whole breast/chest wall plus regional nodes, between patients treated with 3D-CRT and VMAT, and between patients treated with and without tumor bed boost radiotherapy. A Cox proportional hazards regression model was used for univariate and multivariate analysis to investigate risk factors for RIRF among clinical factors and imaging parameters. Continuous variables were categorized into two groups based on the optimal cut-off values determined using the maximum chi-square test. Variables with statistical significance in the univariate analysis were included in the multivariate analysis. For each multivariate analysis model, we estimated Harrell’s concordance index (C-index). The Kaplan–Meier method was used to estimate the cumulative incidence of RIRF according to each imaging parameter. Statistical analyses were performed using MedCalc Statistical Software version 20.011 (MedCalc Software Ltd., Ostend, Belgium) and R software version 4.0.5 (The R Foundation for Statistical Computing, Vienna, Austria). Statistical significance was set at *p* values < 0.05.

## 3. Results

### 3.1. Patient Characteristics

Table 1 presents the baseline characteristics of the enrolled patients. Among 510 patients, 242 (47.5%) were postmenopausal at the time of breast cancer diagnosis, and 173 (33.9%) were overweight or obese (body mass index ≥25.0 kg/m2) based on the World Health Organization definition. Overall, 115 patients (22.6%) had abnormal bone mineral density; among them, 33 (6.5%) had osteoporosis. Regarding radiotherapy, 73 patients (14.3%) were treated with radiation of the whole breast/chest wall plus regional nodes, and 445 patients (87.3%) were treated with the tumor bed boost. Moreover, among the patients, 9 patients (1.8%) were treated with VMAT.

The median clinical follow-up duration of the enrolled patients was 58.8 months (range, 25.1–116.6 months). Follow-up bone scans revealed benign rib fractures and RIRFs in 132 patients (25.9%) and 92 patients (18.0%), respectively. Moreover, RIRFs were found on bone scans performed at a median of 20.0 months (range, 4.8–74.0 months) after the radiotherapy.

### 3.2. Comparisons of Imaging Parameters

All seven imaging parameters were compared according to the RIRF, radiation field, radiotherapy technique, and tumor bed boost (Table 2). Compared with patients without RIRF, those with RIRF had significantly lower values of tumor-rib distance and significantly higher values of V20, V30, and V40 (*p* < 0.05 for all). Moreover, compared with patients without RIRF, those with RIRF had a tendency with borderline significance for lower values of postoperative chest wall thickness (*p* = 0.072) and higher values of V45 (*p* = 0.068) and V50 (*p* = 0.078). For radiation filed, patients who were irradiated only in the whole breast had significantly higher values of postoperative chest wall thickness and significantly lower values of V20, V30, V40, V45, and V50 than those who were irradiated in the whole breast/chest wall plus regional nodes, respectively (*p* < 0.05 for all). However, there were no significant differences in the tumor-rib distance according to the radiation field (*p* > 0.05). For radiotherapy technique, patients treated with VMAT had significantly higher values of V20 and V30 than those treated with 3D-CRT (*p* < 0.05). Regarding tumor bed boost, patients treated with tumor bed boost radiotherapy had significantly higher values of post-operative chest wall thickness than others (*p* = 0.002), but no significant differences were shown for all other imaging parameters (*p* > 0.05).

### 3.3. Risk Factors for RIRF

The relationship between the incidence of RIRF and the seven imaging parameters was assessed using a Cox regression analysis. All continuous variables were dichotomized using optimal cut-off values determined by the maximally selected chi-square test, which was 60 years for age, 50 kg for weight, 1.3 cm for tumor-rib distance, 3.6 cm for post-operative chest wall thickness, 45.1 cm^3^ for V20, 41.7 cm^3^ for V30, 40.8 cm^3^ for V40, 28.2 cm^3^ for V45, and 10.2 cm^3^ for V50. In the univariate analysis, patients with high values of tumor-rib distance and postoperative chest wall thickness had a significantly lower risk of RIRF than those with low values (*p* < 0.05 for both). Contrastingly, patients with high values of V20, V30, V40, V45, and V50 had significantly higher risks of RIRF than those with low values (*p* < 0.05 for all; Table 3). Among the clinical factors, bone mineral density, surgery type, and radiation field were significantly associated with the risk of RIRF, with an increased risk of RIRF in patients with osteoporosis, patients who underwent total mastectomy, and patients whose radiation field included regional nodes (*p* < 0.05 for all, Table 3).

**Figure 2:**
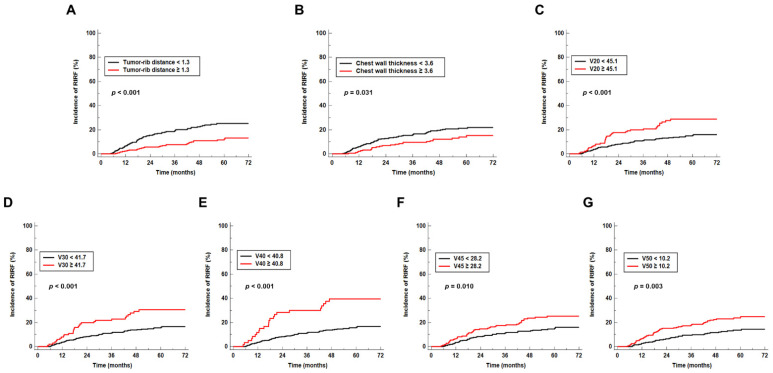
Cumulative incidence of radiation-induced rib fracture (RIRF) according to tumor-rib distance (**A**), postoperative chest wall thickness (**B**), V20 (**C**), V30 (**D**), V40 (**E**), V45 (**F**), and V50 (**G**).

The Kaplan–Meier analysis revealed that patients with high values of tumor-rib distance (11.5%) and postoperative chest wall thickness (14.0%) had a significantly lower five-year cumulative incidence of RIRF than those with low values of tumor-rib distance (25.2%; *p* < 0.001) and postoperative chest wall thickness (21.2%; *p* = 0.031) (Figure 2A,B). Additionally, patients with high values of V20, V30, V40, V45, and V50 showed a significantly higher five-year cumulative incidence of RIRF than those with low values (28.8% vs. 14.9%, *p* < 0.001 for V20; 30.6% vs. 15.6%, *p* < 0.001 for V30, 39.5% vs. 15.7%, *p* < 0.001 for V40; 25.2% vs. 14.6%, *p* = 0.010 for V45; 23.8% vs. 13.6%, *p* = 0.003 for V50; Figure 2C–G).

Bone mineral density, surgery type, radiation field, and all seven imaging parameters, which were statistically significant in the univariate analysis, were selected for the multivariate analysis (Table 4). Since there were significant positive correlations among V20, V30, V40, V45, and V50 (*p* < 0.001 and correlation coefficient >0.500 for all), those five volumetric imaging parameters were assessed in separate models. In multivariate analysis, osteoporosis, radiation field, tumor-rib distance, V20, V30, V40, V45, and V50 were significant risk factors for RIRF (*p* < 0.05). Contrastingly, postoperative chest wall thickness lacked statistical significance in all five multivariate models (*p* > 0.05). Among the five multivariate models, the model with V40 showed the highest C-index value (0.702), followed by the models with V30 (0.694), V20 (0.689), and V45 and V50 (0.684 for both).

## 4. Discussion

Radiation induces bone damages in several ways [20,21]. Bone irradiation decreases the number of osteoblasts, which subsequently decreases collagen production and alkaline phosphatase activity [22]. Since collagen and alkaline phosphatase are crucially involved in the process of bone mineralization, their decrease causes osteopenia [20]. In addition, radiation directly induces atrophic changes in the bone by reducing the amount of calcium and phosphorus [20]. Moreover, radiation causes vascular injuries such as obliterative endarteritis and periarteritis in the bone [20,23]. These inflammatory changes in the vascular structure can lead to atherosclerosis formation and vessel occlusion, which affect blood flow to the bone [12,20,23]. Given these damages, irradiated bones are fragile to the fracture; therefore, in patients with thoracic malignant diseases, follow-up examination after radiotherapy often reveals RIRF [9,21,24]. In our study, 18.0% of patients with breast cancer presented with RIRF after adjuvant radiotherapy. The incidence of RIRF in our study was fairly higher than that in previous studies on patients with breast cancer, which ranged from 1.0% to 3.8% [4,5,6]. However, these previous studies detected RIRF using an X-ray or CT scan, and other previous studies using bone scintigraphy to detect RIRF reported an incidence rate of 12.9–18.5%, which is similar to the incidence rate in our study [9,10,12]. Since bone scintigraphy has a high sensitivity for detecting fractures, and patients with RIRF often lack symptoms, a significant proportion of patients with breast cancer undergoing adjuvant radiotherapy, which could be higher than the expected proportion, might experience RIRF during follow-up without clinical recognition [9,12].

In our study, multivariate analysis revealed that tumor-rib distance was associated with the risk of RIRF. Regarding postoperative radiotherapy for breast cancer, boost radiotherapy to the tumor bed is commonly administered to most patients who undergo breast-conserving surgery, which thereby led to a high post-operative chest wall thickness in patients treated with tumor bed boost radiotherapy. Furthermore, based on histopathological results, some patients who undergo complete mastectomy may receive boost radiotherapy. Therefore, the tumor bed receives a higher radiation dose than other breast tissue. As mentioned above, the tumor-rib distance was defined as the minimum distance between the tumor margin and rib. In other words, it could be assumed that the shorter the tumor-rib distance, the higher dose is irradiated to the rib, resulting in more RIRF occurrence. In a previous study on patients with breast cancer [9], boost radiotherapy was not a significant risk factor for RIRF, as shown in our study; however, the authors discussed that RIRF could have significant association with the tumor-rib distance. Another previous study on patients who underwent pulmonary hypofractionated stereotactic body radiotherapy reported that a tumor-rib distance less than 2.0 cm was the only significant risk factor for RIRF [13]. Unlike our study, this previous study defined the tumor-rib distance as the minimum distance between the radiation isocenter and the rib; nonetheless, it also showed that the tumor-rib distance is negatively correlated with the risk of RIRF, similar to the results of our study.

Dose-dependent rib volume parameters, including V20, V30, and V40, were significantly correlated with the risk of RIRF in the present study. Several studies have investigated the association of total radiation dose or fraction size with the risk of RIRF [8,9]. However, to our knowledge, no studies have assessed the association between the risk of RIRF and the absolute rib volume receiving a certain radiation dose of radiation in patients with breast cancer receiving conventional standard radiotherapy. A previous study on lung cancer reported differences in V20, V30, and V40 between fractured and unfractured ribs [13]. Since this previous study investigated hypofractionated stereotactic body radiotherapy, it could not be directly compared with our study. However, it would be suggested that the absolute rib volume receiving a certain radiation dose can affect the RIRF. In a study on patients with breast cancer who underwent accelerated partial breast irradiation, which is a type of hypofractionated radiotherapy for treating partial breast, the RIRF incidence rate was significantly lower (0.5%) than that in previous studies on whole breast radiotherapy [4,25]. Although these studies were performed in patient populations receiving hypofractionated radiotherapy, the radiation field size was directly correlated with the irradiated absolute rib volume and the risk of RIRF. Therefore, these results imply that a dose-dependent rib volume can be an important risk factor for RIRF.

In our study, enrolled patients were treated with two different radiotherapy techniques, 3D-CRT and VMAT. On comparisons of imaging parameters, patients treated with VMAT showed significantly higher values of V20 and V30, suggesting that dose-dependent rib volumes might be different according to radiotherapy techniques. Although the radiotherapy technique did not show any significant association with the risk of RIRF, because only 1.8% of patients in our study were treated with VMAT, further studies would be needed to assess the relationships of radiotherapy techniques with dose-dependent rib volumes and the risk of RIRF in patients with breast cancer.

To reduce RIRF, it is necessary to consider risk factors for each patient, including tumor-rib distance, and, additionally, precision radiotherapy techniques that allow reduction of the dose-dependent rib volume should be performed for patients at a high risk of RIRF incidence. Among them, one of the approaches for reducing the irradiation dose to the rib is radiotherapy in a prone, rather than supine, position. Treatment in a prone position allows the breast tissue to sag down, which increases the tumor-rib distance, and, additionally, a prone position reduces the absolute rib volume receiving a certain radiation dose. Notably, a study of patients who underwent breast radiotherapy in the prone position reported no RIRF occurrence [26]. However, a prone position is less effective for chest wall irradiation in patients without remnant breast tissue and is unsuitable for treating advanced-stage patients requiring the inclusion of regional lymph nodes. Therefore, the prone position could be selectively applied to early-stage patients and relatively large breast sizes.

The deep-inspiration breath-hold (DIBH) technique is another method for reducing the irradiation dose to the ribs. To our knowledge, no study has assessed the RIRF incidence in patients with breast cancer treated using the DIBH technique. However, DIBH is known to reduce the irradiated dose to the ipsilateral lung through chest wall expansion [27,28,29]. Since the ipsilateral lung is located just behind the ribs, the reduced irradiated dose to the lung suggests a decreased irradiated dose to the ribs. In addition, when the chest wall expands, the rib spacing widens; however, the treatment target does not significantly change; accordingly, there is an expected reduction in the rib volume affected by radiation at a specific dose. In the previous studies [27,28], the dose to the ipsilateral lung was more effectively reduced in patients treated with regional lymph node irradiation. This could allow dose reduction to the ribs in radiotherapy for patients with advanced-stage breast cancer. Future studies that investigate the clinical role of DIBH for reducing RIRF in patients with breast cancer are required.

In addition, discussions are needed in selecting patients who should include ribs after total mastectomy. Previously, in patients with total mastectomy, the treatment target was delineated including ribs generally. However, this contouring guideline basically increases the volume of the ribs exposed to radiation. The most common patterns of local recurrence after total mastectomy are skin and subcutaneous tissues anterior to pectoralis musculature, and they are rarely in the ribs [30]. Since routine inclusion of the ribs and intercostal muscles in all patients can lead to increased heart and lung toxicities, discussion of the patient group to be treated by inclusion of the ribs is needed.

This study had some limitations. First, this was a single-center retrospective study, which might have led to selection bias. Second, since the diagnosis of RIRF was determined using bone scintigraphy and follow-up imaging examinations, the incidence of RIRF in our study could have been overestimated [9].

## 5. Conclusions

In the present study, we found that the tumor-rib distance and dose-dependent rib volumes were independent risk factors for RIRF in patients with breast cancer, along with osteoporosis and the radiation field. Patients with higher values of the tumor-rib distance showed a significantly lower risk of RIRF than others, whereas patients with high values of rib volumes in the radiation field had a significantly higher risk of RIRF than those with low values. In patients with breast cancer who received adjuvant radiotherapy, restriction of the absolute rib volumes in the radiation field should be considered to reduce the risk of RIRF.

## Figures and Tables

**Table 1:** Baseline characteristics of enrolled patients (*n* = 510).

Characteristics		Number of Patients (%)
Age (years)		49 (25–83) *
Weight (kg)		58 (38–90) *
Body mass index (kg/m^2^)		23.4 (15.8–35.2) *
Obesity	Underweight/normal	337 (66.1%)
	Overweight/obesity	173 (33.9%)
Menopausal status	Premenopausal	268 (52.5%)
	Postmenopausal	242 (47.5%)
Bone mineral density	Normal	395 (77.5%)
	Osteopenia	82 (16.1%)
	Osteoporosis	33 (6.5%)
Histopathology	Ductal carcinoma in situ	56 (11.0%)
	Intraductal carcinoma	430 (84.3%)
	Intralobular carcinoma	10 (2.0%)
	Mucinous carcinoma	6 (1.2%)
	Papillary carcinoma	4 (0.8%)
	Others	4 (0.8%)
T stage	Tis, T1–T2	493 (96.7%)
	T3–T4	17 (3.3%)
N stage	N0	375 (73.5%)
	N1–N3	135 (26.5%)
Surgery type	Breast-conserving surgery	467 (91.6%)
	Total mastectomy	43 (8.4%)
Chemotherapy	No	189 (37.1%)
	Yes	321 (62.9%)
Hormone therapy	No	115 (22.5%)
	Tamoxifen	243 (47.6%)
	Aromatase inhibitor	152 (29.8%)
Trastuzumab	No	444 (87.1%)
	Yes	66 (12.9%)
Radiotherapy technique	Three-dimensional conformal radiotherapy	501 (98.2%)
	Volumetric modulated arc therapy	9 (1.8%)
Radiation field	Whole breast	437 (85.7%)
	Whole breast/chest wall + regional nodes	73 (14.3%)
Tumor bed boost	No	65 (12.7%)
	Yes	445 (87.3%)
Tumor-rib distance (cm)		1.3 (0.0–6.7) *
Post-operative chest wall thickness (cm)		3.1 (0.6–8.0) *
V20 (cm^3^)		35.6 (5.2–159.4) *
V30 (cm^3^)		32.1 (4.0–132.5) *
V40 (cm^3^)		28.8 (2.7–91.2) *
V45 (cm^3^)		25.1 (1.4–64.3) *
V50 (cm^3^)		9.8 (0.0–57.4) *

* Median (range) V20, absolute rib volumes receiving more than 20 Gy; V30, absolute rib volumes receiving more than 30 Gy; V40, absolute rib volumes receiving more than 40 Gy; V45, absolute rib volumes receiving more than 45 Gy; V50, absolute rib volumes receiving more than 50 Gy.

**Table 2:** Comparison of tumor-rib distance, post-operative chest wall thickness, and absolute rib volumes receiving more than 20 Gy (V20), 30 Gy (V30), 40 Gy (V40), 45 Gy (V45), and 50 Gy (V50) according to the RIRF, surgery type, and radiation field.

**Imaging Parameters**	**RIRF**			**Radiation Field**		
	**No RIRF** **(*n* = 418)**	**RIRF** **(*n* = 92)**	***p*-Value**	**Whole Breast** **(*n* = 437)**	**Whole Breast/Chest Wall + Regional Nodes** **(*n* = 73)**	***p*-Value**
Tumor-rib distance (cm)	1.5 ± 0.9	1.2 ± 0.6	<0.001	1.5 ± 0.8	1.4 ± 0.7	0.312
Post-operative chest wall thickness (cm)	3.3 ± 1.2	3.1 ± 1.3	0.072	3.3 ± 1.2	2.8 ± 1.5	0.008
V20 (cm^3^)	38.3 ± 15.8	44.3 ± 24.6	0.026	36.2 ± 13.3	58.5 ± 27.5	<0.001
V30 (cm^3^)	33.1 ± 11.8	38.0 ± 19.0	0.021	31.6 ± 10.1	48.5 ± 20.5	<0.001
V40 (cm^3^)	29.5 ± 9.9	32.4 ± 13.3	0.044	28.4 ± 9.0	39.5 ± 13.9	<0.001
V45 (cm^3^)	25.5 ± 8.7	27.9 ± 11.6	0.068	24.8 ± 8.0	33.0 ± 12.9	<0.001
V50 (cm^3^)	11.0 ± 9.2	13.1 ± 10.7	0.078	10.4 ± 8.2	17.5 ± 13.7	<0.001
**Imaging parameters**	**Radiotherapy technique**			**Tumor bed boost**		
	**3D-CRT** **(*n* = 501)**	**VMAT** **(*n* = 9)**	***p*-value**	**No** **(*n* = 65)**	**Yes** **(*n* = 445)**	***p*-value**
Tumor-rib distance (cm)	1.5 ± 0.8	1.2 ± 0.3	0.298	1.5 ± 0.8	1.5 ± 0.8	0.802
Post-operative chest wall thickness (cm)	3.3 ± 1.2	1.5 ± 1.2	0.383	2.7 ± 1.5	3.4 ± 1.2	0.002
V20 (cm^3^)	38.8 ± 17.3	71.7 ± 17.9	<0.001	45.1 ± 27.9	38.5 ± 15.9	0.305
V30 (cm^3^)	33.7 ± 13.3	48.9 ± 15.9	0.001	38.7 ± 21.3	33.3 ± 11.8	0.212
V40 (cm^3^)	29.9 ± 10.5	35.8 ± 15.1	0.320	32.8 ± 14.8	29.6 ± 9.8	0.259
V45 (cm^3^)	25.9 ± 9.2	30.2 ± 15.7	0.776	27.6 ± 12.3	25.7 ± 8.8	0.598
V50 (cm^3^)	11.3 ± 9.4	17.4 ± 14.4	0.099	13.9 ± 13.0	11.0 ± 8.8	0.230

RIRF, radiation-induced rib fracture; 3D-CRT, three-dimensional conformal radiotherapy; VMAT, volumetric modulated arc therapy.

**Table 3:** Univariate analysis of risk factors of radiation-induced rib fractures.

Variables		*p*-Value	Hazard Ratio	95% Confidence Interval
Age (<60 years vs. ≥60 years)		0.215	1.37	0.83–2.25
Weight (<50 kg vs. ≥50 kg)		0.123	1.68	0.87–3.23
Obesity (underweight/normal vs. overweight/obesity)		0.461	0.85	0.54–1.32
Menopausal status (pre- vs. post-)		0.264	1.26	0.84–1.90
Bone mineral density (normal vs.)	Osteopenia	0.410	1.25	0.73–2.14
	Osteoporosis	0.006	2.44	1.28–4.62
T stage (Tis, T1–T2 vs. T3–T4)		0.435	1.49	0.55–4.06
N stage (N0 vs. N1–N3)		0.072	1.49	0.97–2.29
Surgery type (breast-conserving surgery vs. total mastectomy)		0.004	2.33	1.32–4.11
Chemotherapy (no vs. yes)		0.462	1.17	0.76–1.80
Hormone therapy (no vs.)	Tamoxifen	0.218	0.72	0.42–1.22
	Aromatase inhibitor	0.714	1.11	0.64–1.90
Trastuzumab (no vs. yes)		0.184	0.61	0.30–1.26
Radiotherapy technique (3D-CRT vs. VMAT)		0.0820	2.89	0.91–9.20
Radiation field (whole breast vs. whole breast/chest wall + regional nodes)		<0.001	2.53	1.60–4.01
Tumor bed boost (no vs. yes)		0.576	0.85	0.47–1.52
Tumor-rib distance (<1.3 cm vs. ≥1.3 cm)		<0.001	0.43	0.28–0.67
Post-operative chest wall thickness (<3.6 cm vs. ≥3.6 cm)		0.033	0.61	0.38–0.96
V20 (<45.1 cm^3^ vs. ≥45.1 cm^3^)		0.008	2.05	1.35–3.12
V30 (<41.7 cm^3^ vs. ≥41.7 cm^3^)		<0.001	2.12	1.36–3.29
V40 (<40.8 cm^3^ vs. ≥40.8 cm^3^)		<0.001	2.95	1.84–4.72
V45 (<28.2 cm^3^ vs. ≥28.2 cm^3^)		0.011	1.70	1.12–2.56
V50 (<10.2 cm^3^ vs. ≥10.2 cm^3^)		0.004	1.86	1.23–2.84

3D-CRT, three-dimensional conformal radiotherapy; VMAT, volumetric modulated arc therapy; V20, absolute rib volumes receiving more than 20 Gy; V30, absolute rib volumes receiving more than 30 Gy; V40, absolute rib volumes receiving more than 40 Gy; V45, absolute rib volumes receiving more than 45 Gy; V50, absolute rib volumes receiving more than 50 Gy.

**Table 4:** Multivariate analysis of risk factors of radiation-induced rib fractures.

Variables		Model with V20		Model with V30		Model with V40		Model with V45		Model with V50	
		*p*-Value	Hazard Ratio (95% CI)	*p*-Value	Hazard Ratio (95% CI)	*p*-Value	Hazard Ratio (95% CI)	*p*-Value	Hazard Ratio (95% CI)	*p*-Value	Hazard Ratio (95% CI)
Bone mineral density	Osteopenia	0.396	1.26 (0.74–2.17)	0.406	1.26 (0.73–2.16)	0.365	1.29 (0.75–2.21)	0.244	1.39 (0.80–2.44)	0.396	1.26 (0.74–2.17)
	Osteoporosis	0.004	2.64 (1.37–5.11)	0.003	2.77 (1.43–5.37)	0.002	2.91 (1.50–5.62)	0.002	2.87 (1.47–5.26)	0.005	2.57 (1.33–4.95)
Surgery type		0.562	1.22 (0.62–2.42)	0.538	1.24 (0.63–2.43)	0.459	1.29 (0.66–2.54)	0.423	1.32 (0.67–2.60)	0.645	1.18 (0.59–2.34)
Radiation field		0.049	1.79 (1.00–3.20)	0.071	1.73 (0.95–3.13)	0.123	1.59 (0.88–2.86)	0.023	1.91 (1.09–3.32)	0.005	2.18 (1.26–3.78)
Tumor-rib distance		0.001	0.46 (0.29–0.74)	0.001	0.46 (0.29–0.73)	<0.001	0.45 (0.28–0.71)	0.001	0.46 (0.29–0.73)	0.002	0.48 (0.30–0.77)
Post-operative chest wall thickness		0.374	0.80 (0.49–1.31)	0.305	0.78 (0.48–1.26)	0.478	0.84 (0.51–1.37)	0.457	0.83 (0.51–.35)	0.517	0.85 (0.52–1.39)
V20		0.029	1.68 (1.06–2.69)	-	-	-	-	-	-	-	-
V30		-	-	0.035	1.73 (1.04–2.89)	-	-	-	-	-	-
V40		-	-	-	-	<0.001	2.51 (1.47–4.29)	-	-	-	-
V45		-	-	-	-	-	-	0.028	1.65 (1.06–2.58)	-	-
V50		-	-	-	-	-	-	-	-	0.039	1.58 (1.02–2.45)

CI, confidence interval; V20, absolute rib volumes receiving more than 20 Gy; V30, absolute rib volumes receiving more than 30 Gy; V40, absolute rib volumes receiving more than 40 Gy; V45, absolute rib volumes receiving more than 45 Gy; V50, absolute rib volumes receiving more than 50 Gy.

## Data Availability

The datasets used and/or analyzed during the current study are available from the corresponding author on reasonable request.
